# Supplementary material for: Alpha-synuclein overexpression triggers divergent cellular responses and post-translational modifications in SH-SY5Y and ReNcell VM models
Source: Cell Mol Life Sci. 2026 Feb 21;83(1):123. doi: 10.1007/s00018-026-06126-z (PMC12932749; doi:10.1007/s00018-026-06126-z)
Supplement: Supplementary file 1 — Supplementary Material 1 [file 18_2026_6126_MOESM1_ESM.docx]

**Figure S1. Characterization of eGFP-expressing control cell lines and their mitochondrial phenotype** (A) Representative flow cytometry (FACS) plots showing the gating strategy used to isolate GFP-positive and GFP-negative populations in SH-SY5Y and ReNcell VM cells. Among GFP-positive cells, subpopulations were further stratified into “low” and “high” expression groups based on GFP fluorescence intensity, using the same thresholds applied for α-syn low (L) and high (H) overexpression lines. (B) Representative immunofluorescence images of GFP signal in sorted cells, co-stained with TOM20 to assess the impact of GFP expression on mitochondrial morphology. Images were acquired under identical exposure conditions for each cell line. (C) Western blot analysis evaluating GFP protein migration and mitochondrial mass using TOM20 as a mitochondrial marker. HSP90 was used as a loading control. In SH-SY5Y cells, only the high GFP expression condition (GFP H) was included for protein-level validation. (D–E) Functional assessment of cell viability in GFP-expressing cells using Resazurin (D) and MTT (E) assays, normalized to non-transduced controls. (F) Seahorse XF Cell Mito Stress Test evaluating mitochondrial function in GFP control lines. Oxygen consumption rate (OCR) was measured over time and analyzed for basal respiration and maximal respiration capacity. Group comparisons were performed using one-way ANOVA followed by Dunnett’s post hoc test for multiple comparisons. Statistical significance is indicated as p < 0.05 (*), p < 0.01 (**), and p < 0.001(***); non-significant p > 0.05.

Note: SH-SY5Y GFP low group was not analyzed by Western blot but was included in all other functional assays.


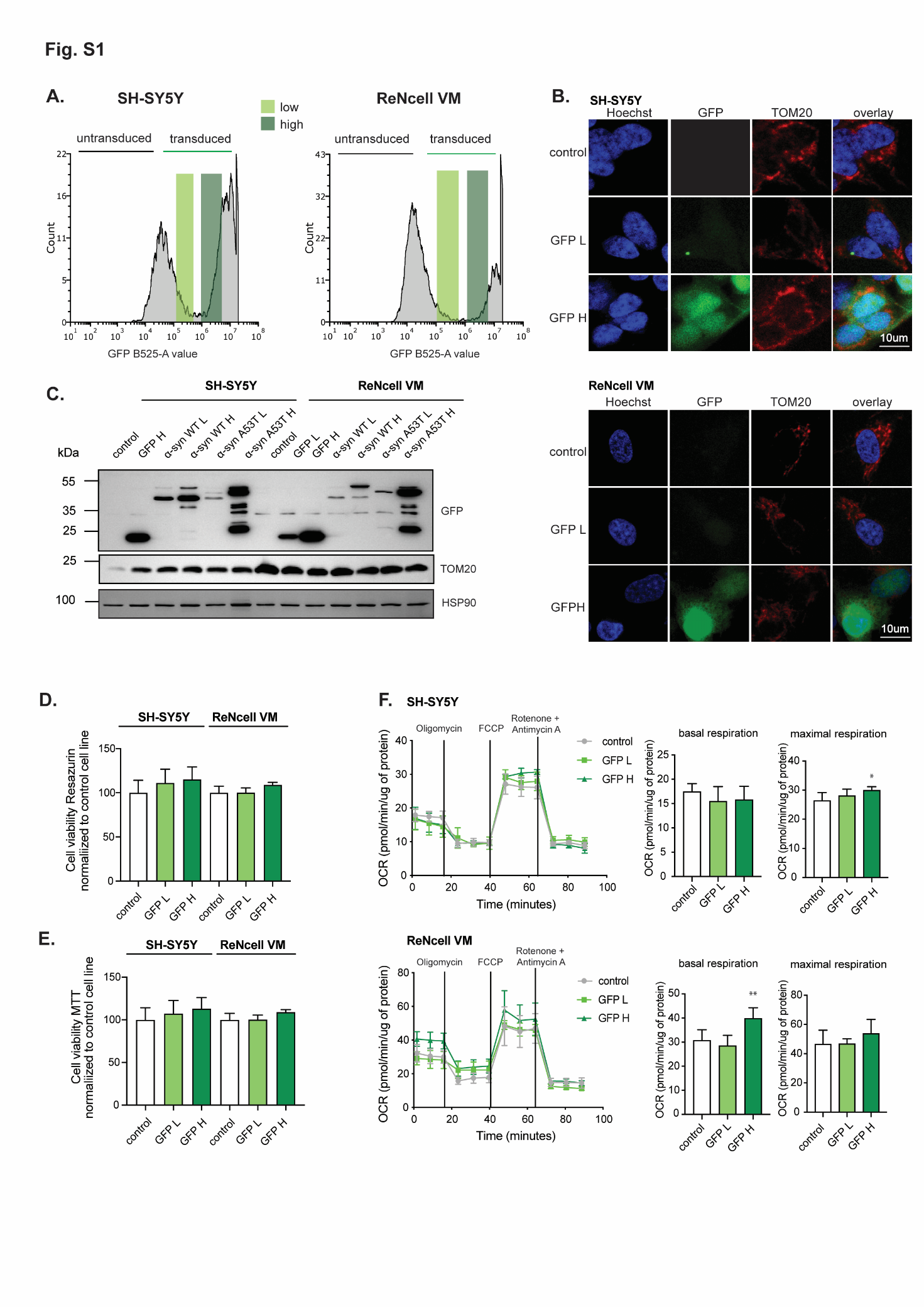

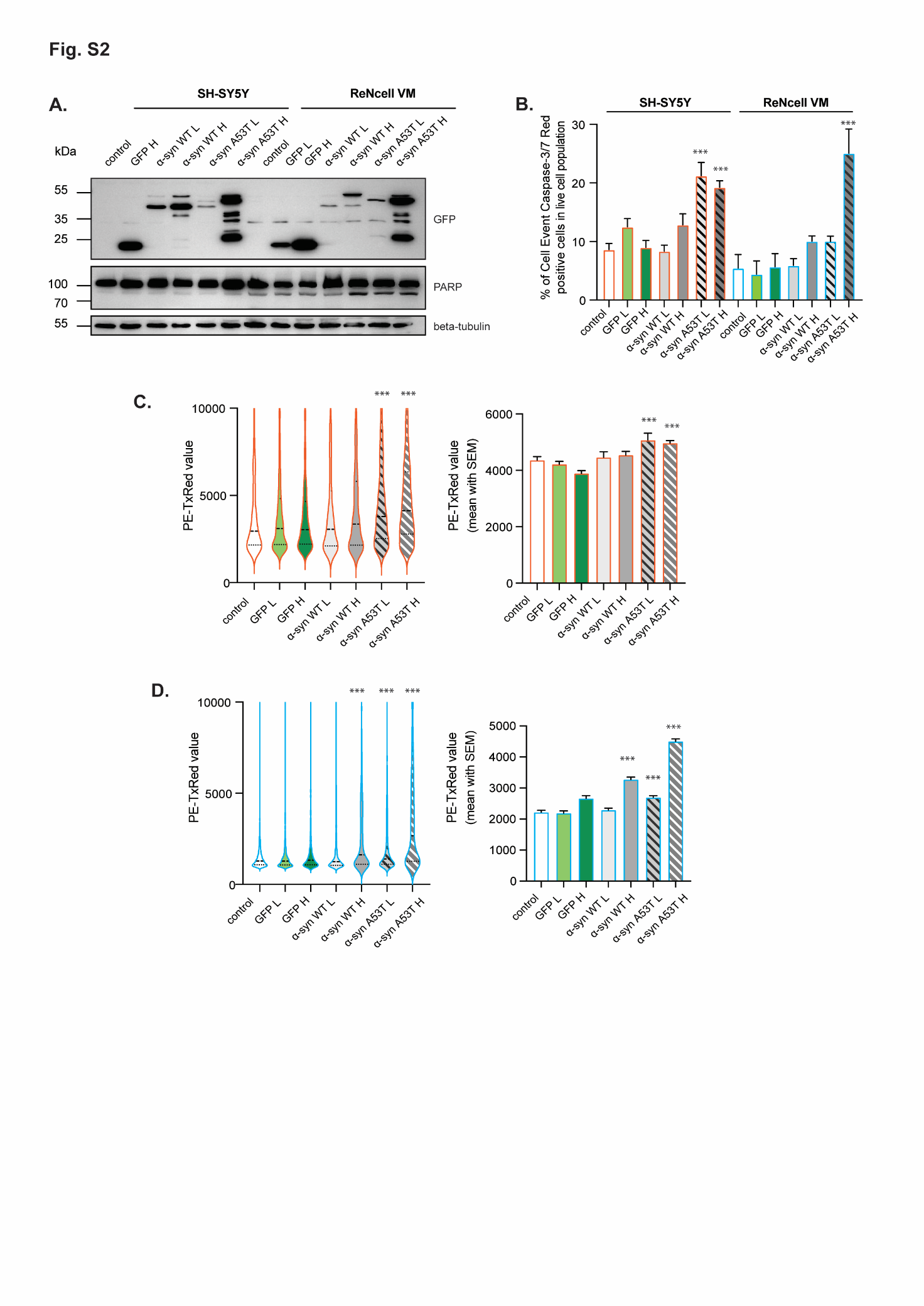


**Figure S2. Assessment of apoptosis in α-syn and GFP-expressing cell lines.** (A) Western blot analysis of full-length and cleaved poly(ADP-ribose) polymerase (PARP) in SH-SY5Y and ReNcell VM cells expressing wild-type or A53T α-syn, as well as GFP-only controls. The appearance of a lower molecular weight band indicates the presence of cleaved PARP, a marker of apoptosis. β-tubulin was used as a loading control. (B) Quantification of apoptosis using CellEvent™ Caspase-3/7 Red detection reagent. The percentage of apoptotic cells was determined by flow cytometry across all experimental conditions. (C–D) Quantitative flow cytometric analysis of Caspase-3/7 Red fluorescence intensity in SH-SY5Y (C) and ReNcell VM (D) cells. Violin plots depict the distribution of signal intensities within the population, while accompanying bar graphs represent the mean fluorescence intensity (MFI) of the apoptotic marker. Column graphs show the mean ± SEM as an alternative presentation of changes in staining intensity. Group comparisons were performed using one-way ANOVA with Dunnett’s post hoc test for multiple comparisons. Statistical significance is indicated as p < 0.05 (*), p < 0.01 (**), and p < 0.001(***); non-significant p > 0.05.
